# Associations between mental health & substance use treatment and alcohol use progression and recovery among US women drinkers

**DOI:** 10.1371/journal.pone.0306820

**Published:** 2024-07-08

**Authors:** Andrea S. Young, Beth A. Reboussin, Kira Riehm, Ramin Mojtabai, Kerry M. Green, Emily T. O’Gorman, Ryoko Susukida, Masoumeh Amin-Esmaeili, Rosa M. Crum

**Affiliations:** 1 Department of Psychiatry, Johns Hopkins University School of Medicine, Baltimore, Maryland, United States of America; 2 Department of Mental Health, Johns Hopkins Bloomberg School of Public Health, Baltimore, Maryland, United States of America; 3 Department of Biostatistics and Data Science, Wake Forest School of Medicine, Winston-Salem, North Carolina, United States of America; 4 Department of Behavioral and Community Health, University of Maryland School of Public Health, College Park, Maryland, United States of America; 5 Department of Epidemiology, Johns Hopkins Bloomberg School of Public Health, Baltimore, Maryland, United States of America; University of Jyvaskyla, FINLAND

## Abstract

**Background:**

Alcohol use has profound public health impact on women; however, modifiable factors that may influence alcohol use progression/recovery, including health service utilization, are understudied in women.

**Objective:**

To investigate the association between mental health (MH) and substance use (SU) treatment with alcohol use progression and recovery among women who currently use alcohol or have in the past.

**Methods:**

This study is a secondary data analysis of prospective data from waves 1 (2001–2002) and 2 (2004–2005) of the National Epidemiologic Survey on Alcohol and Related Conditions (NESARC; a US-nationally representative sample of adults). The analytic sample was limited to women who reported past or current alcohol use at wave 1 (N = 15,515). Latent transition analysis (LTA) examined whether receiving SU/MH treatment in the year prior to wave 1 was associated with transitioning between three empirically-derived stages of alcohol involvement (no, moderate, and severe problems classes), between Waves 1 and 2 adjusting for possible confounders using propensity score weight.

**Results:**

Compared to White female drinkers, female drinkers who were from Black, Hispanic, or other races were less likely to receive SU/MH treatment (*p*-values ≤. 001). SU/MH treatment in the year prior to wave 1 was associated with transitioning from the moderate problems class to the no problems class between Waves 1 and 2 (*p*-value = .04).

**Conclusion:**

Receipt of SU or MH treatment among women, was associated with a higher likelihood of remission from moderate alcohol use problems to no problems over time. Future research, including investigation into treatment characteristics (e.g., frequency, duration, type) should further explore why women initially experiencing severe alcohol use problems did not experience similar remission.

## Introduction

The individual and public health costs of problematic alcohol use are high. In the United States, recent report indicates the average annual deaths due to alcohol use was over 140,000 during 2015–2019 [1], an increase from 95,000 deaths annually reported in 2010 [2]. The estimated costs from alcohol use-associated loss of work productivity, healthcare, criminal justice, and motor vehicle accidents were $249 billion in 2010 [2]. Alcohol use is associated with risk for injury [3,4], illness [5], and criminal justice system involvement [6], among other negative outcomes [7]. The prevalence of alcohol use and abuse have increased among women in particular since the early 2000s [8]; the rate of alcohol use disorder (AUD) in women increased 84% within this time, compared to a 35% increase in men, reflecting a decreasing gap between sexes in prevalence of AUD [9].

Various AUD treatments, ranging from community-based outpatient programs to inpatient interventions, have demonstrated efficacy [10–13]. However, despite the costly and pernicious outcomes associated with problematic alcohol use, alcohol use treatments are underutilized [14], particularly among women. According to data from the 2021 National Survey on Drug Use and Health, women who met criteria for a past-year AUD are substantially less likely than men to receive any alcohol use treatment (3% vs. 5.6%) [15]. Similarly, data from the National Epidemiologic Survey on Alcohol and Related Conditions (NESARC), a representative sample of US adults, indicate that women with AUD seek any alcohol treatment help less often than men with AUD (10.9% vs 15.7%) [9,16].

Comorbid substance use and psychiatric disorders [17] and more severe AUD presentation both predict treatment utilization [9]. Prior analyses of NESARC data indicate that, among those with AUD at baseline, receiving treatment that included participating in a 12-step program was associated with abstinent recovery (no alcohol use or symptoms) three years later at follow-up [18]. There is also evidence that receiving mental health treatment for a psychiatric comorbidity (e.g., depression and anxiety) can improve not only the psychiatric symptoms but also alcohol and other substance use outcomes both when it is adjunctive [19,20] and when it is the primary intervention [21,22]. However, there is substantial heterogeneity in patterns of progression and remittance in alcohol use [23]. Of note, there is a dearth of research examining AUD treatments in sex- or gender-specific samples or reporting differences based on sex or gender [24,25].

Given the significant consequences of problematic substance use and heterogeneous patterns, it is important to better understand modifiable predictors of change. Health services use, including general mental health treatment and substance use treatment, may be one such predictor that could impact AUD symptoms. However, the impact of treatment in usual care settings on AUD symptoms remains unclear. While observational data, such as that obtained through NESARC, potentially have good external validity for examining the effects of treatment in real world and usual care settings, examining treatment use with observational data can be challenging [26]. Results of analyses of treatment effects on alcohol problems in NESARC have been mixed. For example, one analysis found that treatment seeking is associated with more severe presentations of alcohol use at baseline [27], while others indicated that treatment is associated with abstinence (no alcohol use or symptoms) at follow-up [17,18].

In the current study, we sought to build on this prior work, and prior work from our group that examined the role of mood disorders in transitions in alcohol classes [23], by examining to what extent mental health and substance use treatment utilization are associated with transitions across stages of alcohol involvement among women with current and/or past alcohol use in a population-based sample. We chose to focus on women specifically due to the documented sex differences in treatment seeking and service utilization between sexes, with women receiving substance use treatment less frequently than men overall [17,28]. We hypothesized that having received mental health and/or substance use treatment within the past year at wave 1 would be associated with decreased odds of progressing to a more severe class and increased odds of recovering to a less severe class, relative to not receiving mental health and/or substance use treatment. This current study used 1) rich longitudinal data on substance use, psychiatric illness, and treatment; 2) latent class analysis (LCA) and latent transition analysis (LTA) to examine transitions between qualitatively different groups of current and/or past drinkers; 3) propensity score adjustment to reduce the effects of confounders; and 4) a US-representative sample of adults to further elucidate the role of mental health and substance use treatment in real-world settings on women’s alcohol use progression and recovery.

## Materials and methods

The current study is a secondary data analysis of prospective data from waves 1 (2001–2002) and 2 (2004–2005) of the National Epidemiologic Survey on Alcohol and Related Conditions (NESARC), a nationally representative sample of adults in the US [17,29]. NESARC oversampled Black, Hispanic, and young adult (age 18–24 years) populations, as these populations had been understudied in prior literature [30]. Wave 1 assessed 43,093 participants, aged ≥ 18 years, through face-to-face interviews. Of these, 39,959 remained eligible for wave 2 follow-up (which was scheduled approximately 3 years after the first interview), and 34,653 participated in this follow-up (87% response rate). Participants were ineligible for the wave 2 assessment if they were on active military duty, deported, mentally or physically impaired, or deceased. The current analyses were limited to women who reported any current (at wave 1) or past (prior to wave 1) alcohol use; 15,515 women meeting these criteria had data available at both waves. The original NESARC study through which these data were collected was reviewed and approved by the US Census Bureau and US Office of Management and Budget; participants provided informed consent prior to participating in the original study. The current manuscript utilizes only NESARC’s publicly-available, fully de-identified dataset; the authors of this manuscript did not have access to any participant’s protected health information. Thus, the Johns Hopkins Medicine Institutional Review Board determined the current research did not constitute human subjects research.

### Measures

#### Mental health and substance use

Participants reported symptoms of alcohol use and substance use disorder and their history of treatment for these problems using the Alcohol Use Disorder and Associated Disabilities Interview Schedule–DSM-IV version (AUDADIS-IV) [30]. Specifically, the AUDADIS-IV assesses use of alcohol, nicotine, sedatives, tranquilizers, opiates, stimulants, hallucinogens, cannabis, cocaine, and inhalants/solvents. This assessment included questions about drinking quantity (the number of drinks of any alcohol usually consumed on days when the participant drank alcohol in the past 12 months) and frequency (how often the participant drank in the last 12). The AUDADIS-IV also assessed for major mood, anxiety, attention-deficit/hyperactivity, and personality disorders as well as a family history of mental illness.

#### Latent classes of alcohol use

Similar to our prior work [23], we used AUD symptoms from the AUDADIS-IV as indicators in a latent class analysis (LCA) to identify stages of alcohol use. The eleven indicators included assessed clinical features of DSM-IV alcohol abuse (4 indicators) and dependence (7 indicators). The full list of indicators is available in S1 Table. Transitions across classes between wave 1 and wave 2 were the outcome variables of interest.

#### Treatment

Any past year substance use (SU) or other mental health (MH) treatment (vs. no treatment) reported at wave 1 was the predictor. MH treatment included: talking to a counselor, therapist, doctor, or psychologist; being prescribed medication; visiting an emergency room; and overnight hospitalization. SU treatment included: 12-step programs; private physician, psychiatrist, psychologist, social worker or other professional; rehabilitation programs, family service of other social service; other agency or professional; detoxification ward/clinic; outpatient clinic; emergency room; inpatient ward of a hospital or community mental health program; clergy or religious counselors; employee assistance programs; halfway houses/therapeutic communities; crisis centers; and methadone maintenance.

#### Potential confounders

Demographics confounders measured at wave 1 included age (18–35 years, 36–49 years, 50+ years), race/ethnicity (i.e., non-Hispanic White, non-Hispanic Black, Hispanic, and other race), health insurance (i.e., Medicaid, Medicare, military health care, private health insurance), and education (coded categorically as 12 or more years or less than 12 years).

Clinical confounders were assessed via the AUDADIS and included history of mental health disorders, history of illicit drug or nicotine use disorders, family history of alcohol use disorder, type of treatment at wave 1 (as three different binary variables indicating whether individuals had received a) medical invention, b) participated in a 12-step program, or c) received social services), drinking status (current [at wave 1] or past/lifetime), early drinker status (first drink before age 13 years), number of mental health disorders, number of substance use disorders, drinking quantity (the number of drinks of any alcohol usually consumed on days when the participant drank alcohol in the past 12 months) and frequency (how often the participant drank in the last 12), and lifetime personality disorder. Lastly, the SF-12 Physical and Mental component scores were considered potential confounders and were assessed via the SF-12 Health Survey [31].

### Analytic plan

To describe the sample, we used chi squared tests to determine whether there were differences at baseline in treatment seeking across demographic groups.

In order to identify groups of women with similar patterns of alcohol involvement, we performed LCAs on the DSM-IV alcohol abuse (4 criteria) and dependence criteria (7 criteria) assessed via the AUDADIS-IV. We completed separate LCAs for W1 and W2 [23]. We examined models with increasing numbers of latent classes in order to identify the best fitting, most parsimonious model based on fit indices (Akaike’s information criteria [AIC], Bayesian information criterion [BIC], and the sample-size adjusted BIC [aBIC]), with lower values on these indices indicating better fit [32]. When selecting models, we also considered interpretability and entropy (with values approaching 1 indicating clearer class distinction) to reduce the likelihood of identifying spurious cases [33].

Latent transition analysis (LTA) estimated probabilities of moving from one latent class at W1 to another latent class at W2 relative to the probability of remaining in the same class conditional upon latent class membership at W1 [34,35] accounting for NESARC sampling weights, clustering, and stratification. LTAs captured moving to a more severe stage of alcohol involvement or a less severe stage.

Using a multinomial regression formulation, we first examined cross-sectional associations between latent class membership at wave 1 with past year SU or MH treatment. We then used the LTA models to examine the effect of past year SU or MH treatment on transitions in alcohol involvement symptoms, using a multinomial regression formulation. To account for baseline imbalance in potential confounding variables between those who received and did not received treatment in this non-randomized design, we applied the inverse probability of treatment weighting (IPTW) approach based on propensity scores [36–38]. We computed propensity scores using a logistic regression model in R, with any substance use or other mental health treatment versus no treatment as the independent variable. The effectiveness of IPTW in balancing the composition of the exposure groups was deemed successful after characteristics of the groups were compared before and after applying the weights [38]. Propensity score models adjusted for the confounders listed above. Propensity score weights were then multiplied by the sampling weights in these LTA models in addition to accounting for the other design factors [39]. All LCA and LTA analyses were performed using *Mplus* version 7.0 [40].

## Results

### Characteristics of women who drink by treatment history

Characteristics of past or current drinkers by SU or MH treatment history are presented in Table 1. Women who had not received SU or MH treatment were more likely to be 50 years old or older (p < .001). Women who were non-Hispanic Black, Hispanic, or other races were overrepresented among the group that had not received treatment (p ≤ .001). Specifically, we found 11.9% of white female past or current drinkers engaged with SU or MH treatment, compared to 8.8% of Black, 10.9% of Hispanic, and 9.6% of those of other races/ethnicities female current or lifetime drinkers. Women with a mood disorder, anxiety disorder, nicotine use disorder, or family history of alcoholism were more likely to have received SU or MH treatment (*p* < .01).

**Table 1:** Baseline (wave 1) characteristics of past and current women drinkers (N = 15,320) by a history of SU or MH treatment in the past year at wave 1: NESARC.

Characteristics at baseline	*Total*		Any SU or MH Treatment Past Year Wave 1				
			Absent		Present		*p*-value^a^
	N	%	N	%	N	%	
Age (years): 18–35 36–49 50+	5002 4616 5702	32.9 30.4 36.7	4391 4017 5211	32.3 29.8 37.8	611 599 491	37.6 35.1 27.3	**<0.001**
Race/ethnicity: White Black Hispanic Other	9609 2960 2387 364	77.2 10.5 9.0 3.3	8462 2701 2127 329	76.8 10.7 9.2 3.3	1147 259 260 35	80.7 8.7 7.8 2.8	**<0.001**
Education (years): <12 ≥12	1972 13348	11.2 88.8	1747 11872	11.1 88.9	225 1476	11.6 88.4	0.254
Mood disorder^b^: Major Depressive Disorder Dysthymia Mania Hypomania Any Mood Disorder	3658 970 625 406 4138	23.7 6.0 4.1 2.4 26.6	2457 510 322 313 2820	18.1 3.6 2.4 2.1 20.6	1201 460 303 93 1318	70.8 25.7 17.9 5.5 77.1	**<0.001**
Anxiety disorder^b^: GAD^c^ Panic disorder Social phobia Specific phobia Any Anxiety Disorder	931 870 935 2072 3592	6.2 5.8 6.3 13.7 23.8	515 521 627 1631 2650	4.0 4.8 4.8 12.1 19.8	416 349 308 441 942	24.7 29.8 18.6 26.6 56.4	**<0.001**
Nicotine use disorder Absent Present	12599 2721	81.6 18.4	11510 2109	83.9 16.1	1089 612	62.4 37.6	**<0.001**
Family history of alcoholism: Absent Present	9597 5723	63.2 36.8	8799 4820	65.0 35.0	798 903	47.5 52.5	**<0.001**

Note: Percentages included in the table are column percentages (i.e., the dominator is the number of women who did or did not receive substance use or mental health treatment).

^a^ Rao-Scott chi-square tests of difference.

^b^ Diagnostic categories are not mutually exclusive.

^c^ GAD, generalized anxiety disorder.

#### Latent class analysis

**Fig 1:**
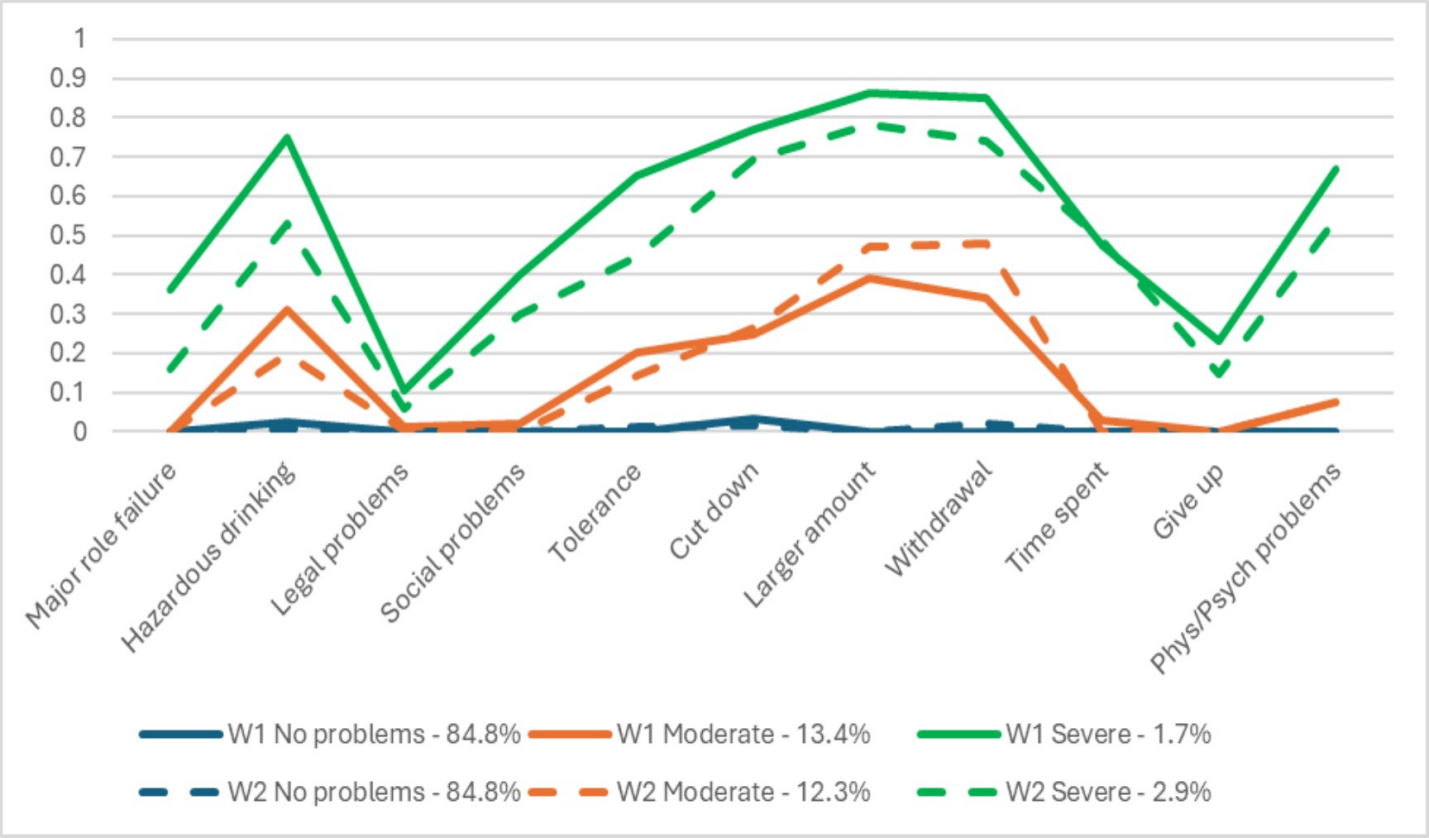
Patterns of alcohol problem classes item loadings across waves from the NESARC waves 1 and 2 among female drinkers.

As shown in Table 2, fit indices, entropy, and interpretability favored a 3-class-solution for alcohol involvement classes. Patterns of item loadings were similar across waves and are depicted in Fig 1. Similar to prior analyses from our group [23], the classes represented alcohol involvement in the past year, with increasing severity across classes: the “no problems” class included drinkers who experienced no or very few AUD symptoms; the “moderate problems” class was characterized by drinking in hazardous situations, drinking larger amounts, and withdrawal symptoms; and the “severe problems” class was characterized by higher item probabilities than the moderate problems class as well as role failure, social problems, tolerance, problems cutting down alcohol consumption, greater time spent getting alcohol and physical and psychiatric problems. The no problems class was the most prevalent (84.8% at both waves), followed by the moderate problems class (13.4% at wave 1 and 12.3% at wave 2); the severe problems class was the least prevalent (1.7% at wave 1 and 2.9% at wave 2).

**Table 2:** Fit indices and entropy for latent class analyses of alcohol use involvement for NESARC Waves 1 and 2.

	No. of Classes	AIC^a^	BIC^b^	a-BIC^c^	Entropy^d^
**Wave 1**	1	46152	46236	46201	1.00
	2	36690	36866	36793	0.94
	3	35845	36114	36002	0.88
	4	35711	36070	35821	0.72
**Wave 2**	1	47056	47140	47105	1.00
	2	37260	37436	37363	0.93
	3	36435	36703	36591	0.87
	4	36400	36759	36610	0.77

^a^Akaike’s information criteria.

^b^Bayesian information criterion.

^c^sample-size adjusted BIC.

^a,b,c^lower values indicate better fit.

^d^values approaching 1 indicate clearer class distinction.

There were small differences in item probabilities across waves, but the overall interpretation of the classes was the same. As such, similar to our prior analyses [23], we imposed measurement invariance across waves in the LTA.

#### Latent transition analyses

**Fig 2:**
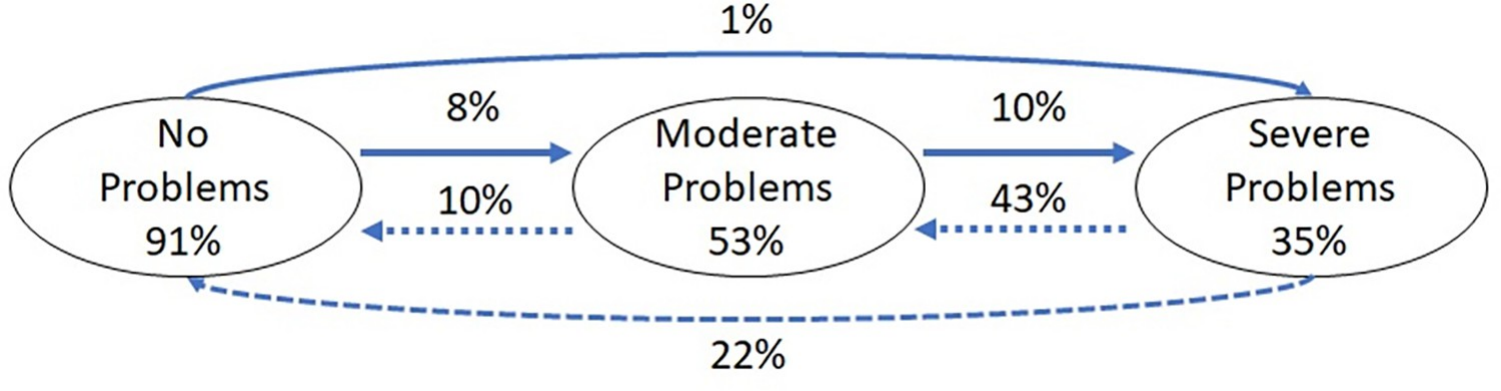
Probabilities of transitioning across stages of alcohol use between NESARC waves 1 and 2. The proportions in each oval represent the probability of remaining in the same class at the 3-year follow-up (e.g., 91% in the “No problems” oval indicates the probability of remaining in the no problems class at wave 2 among those in the no problems class at wave 1). Arrows between ovals depict the probability of transitioning from one group to another between waves 1 and 2. All probabilities are adjusted for sampling weights.

First, we estimated the overall probabilities of transitioning between classes over the two waves (Table 3 and Fig 2). Women who started in the no problems class at wave 1 were most likely to remain in the no problems class at wave 2. Specifically, 91% of those in the no problems class at wave 1 remained in that class at wave 2, whereas 8% of women in the no problem class in wave 1 transitioned to the moderate problems class, and 1% transitioned to the severe class in wave 2. Over half of women (53%) who were in the moderate problems class at wave 1 were in the same class at wave 2. About 10% of women in the moderate class at wave 1 progressed to the severe class at wave 2, while 37% improved and moved to the no problems class at wave 2. Among those who were in the severe problems class at wave 1, 35% continued to have severe problems, 43% transitioned to the moderate class, and 22% transitioned to the no problems class at wave 2.

**Table 3:** Probabilities of transitioning between latent alcohol use classes across NESARC waves 1 and 2 (adjusted for sampling weights).

Class at Wave 1	Class at Wave 2		
	No problems	Moderate	Severe
No problems	0.913	0.079	0.008
Moderate	0.366	0.529	0.105
Severe	0.217	0.436	0.347

Next, we included treatment in the past year as a covariate in the LTA model. As shown in Table 4, women who received SU or MH treatment were more likely to be in the moderate or severe problems class than the no problems class at wave 1. However, when examining transitions in alcohol problems classes from wave 1 to wave 2 (Table 5), receipt of any SU or MH treatment within the past year at wave 1 was associated with being 1.82 times as likely to transition from moderate problems to no problems in an LTA model that adjusted for sampling weights and propensity weights (and, thereby, potential confounders including demographics, history of mood and anxiety disorder, use of illicit drugs and nicotine, and other SU and treatment characteristics) (*p* = .043). Among women classified as having severe problems at wave 1, receiving treatment was associated with being 0.23 times as likely to remit to moderate problems (*p* = .001) in an LTA model adjusting for sampling weights alone; however, this association became nonsignificant once propensity score weights were included in the model (*p* = .439). Receipt of SU or MH treatment was not significantly associated with transitioning from severe problems to no problems. When examining alcohol use progression, having received SU or MH treatment was not associated with transitioning to more severe problem classes.

**Table 4:** Cross-sectional association of health services with alcohol involvement class at baseline: NESARC wave 1 (n = 15,320).

Alcohol Involvement Class	Sampling Weight Adjusted Odds Ratio, p-value	Propensity Score Adjusted Odds Ratio, p-value
No Problems	Reference	
Moderate	**2.30 (1.83, 2.88), p<0.001**	**1.42 (1.01, 1.84), p = 0.007**
Severe	**4.86 (3.68, 6.42), p<0.001**	1.14 (0.74, 1.73), p = 0.558

**Table 5:** Any SU or MH treatment past year wave 1 predicting transitions in stages of alcohol involvement from wave 1 to wave 2 among NESARC past and current women drinkers (n = 15,320).

Alcohol Progression	Row 1: sampling weights Row 2: sampling weights + propensity weights
No Problems to Moderate	0.97 (0.63, 1.47), p = 0.870 1.01 (0.59, 1.74), p = 0.967
No Problems to Severe	1.42 (0.49, 4.07), p = 0.517 0.66 (0.15, 2.84), p = 0.757
Moderate to Severe	1.22 (0.60, 2.50), p = 0.587 1.35 (0.55, 3.28), p = 0.512
**Alcohol Remission**	
Moderate to No Problems	1.52 (0.94, 2.44), p = 0.087 **1.82 (1.02, 3.24), p = 0.043**
Severe to No Problems	1.03 (0.47, 2.28), p = 0.942 1.34 (0.38, 4.75), p = 0.649
Severe to Moderate	**0.23 (0.10, 0.54), p = 0.001** 0.60 (0.16, 2.19), p = 0.439

*Propensity score models adjusted for age, race, education, history of mental health disorders at wave 1 (anxiety, mood), history of substance use disorders at wave 1 (illicit, nicotine), family history of alcoholism, drinking status (current, past/lifetime, none), early drinker, # mental health comorbidities, # substance use disorders, drinking quantity, drinking frequency, insurance coverage at wave 1, SF-12 physical component score, SF-12 mental component score, and lifetime personality disorder.

## Discussion

This study sought to investigate the association of receiving SU or MH treatment with progression and remission across stages of alcohol involvement in a US-representative sample of adult women with past or current alcohol use. The current analyses built on prior analyses establishing latent classes characterized by severity of drinking problems and the role of mood disorders in transitions across classes of alcohol use [23]. Consistent with our prior work, results of the LTA indicate that the no problems class is fairly stable across waves with 91% of women in the no problems class at wave 1 remaining in that class in wave 2 [23]. In general, remission to a less severe class of alcohol involvement was more common than progression to a more serious class. For women who were in the moderate problem class at wave 1, only about 10% transitioned to the severe class, while 37% transitioned to the no problems class. Of the women in the severe class at wave 1, about 65% improved overall and transitioned to a less severe class at wave 2.

We hypothesized that receipt of SU or MH treatment in women would be associated with decreased odds of progression (i.e., decreased likelihood of transitioning to a more severe class of alcohol involvement) and increased odds of remission (i.e., increased likelihood of transitioning to a less severe class of alcohol involvement). Results were partially consistent with these hypotheses. Among female past and current drinkers, those who had reported past year SU or MH treatment at wave 1 of the NESARC were significantly more likely to transition from moderate problems to no problems compared to those who had not received treatment, after adjustment for confounders. Prior clinical trials, reviews, and meta-analyses document the well-established benefits of treatments, both to individuals with alcohol and other substance use problems and to society in general [41–45]. Our findings concerning alcohol remission are largely consistent, suggesting that SU or MH treatment was associated with remitting alcohol involvement across classes in most cases. Of note, among women in the severe problems at wave 1, receiving treatment was associated with decreased likelihood to remit to moderate problems when adjusting for sampling weights alone; however, this association became nonsignificant once propensity score weights were included in the model. This suggests the findings from the sampling-weight-only model are accounted for by demographic variables, family history, and other substance use related factors. Among women in the severe problems group at wave 1, behavioral health treatment was not significantly associated with transitioning to the no problems group. Severe problems may simply be more persistent or challenging to treat. Contrary to our hypothesis, women who had received treatment were not significantly less likely to progress to more severe classes. We adjusted for several factors that might be associated with participants’ treatment status and alcohol involvement, including age, race, education, history of mood or anxiety disorders, history of illicit substance or nicotine use, prior treatment, type of treatment and many other factors. Despite these efforts, the results did not suggest a significant association between SU or MH treatment and decreased symptom progression across the three-year period. Given the well-documented benefits of alcohol and substance use treatment [41–45], this finding is surprising.

This finding may be explained in part by transitions to more severe classes of alcohol involvement within the time period examined being quite uncommon. The vast majority of those with alcohol problems at wave 1 remained in their same class at wave 2 or transitioned to a less severe class, with less than 2% of the overall sample with alcohol problems at wave 1 moving to a more severe class at wave 2. Another potential explanation for these findings is that individuals with more severe, impairing, or worsening problems are more likely to receive treatment compared to those with low-level or developing problems. Our cross-sectional analyses found that using SU and/or MH services in the past year was associated with being in a more severe alcohol use class at wave 1. This suggests that women with more alcohol use problems are most likely to receive substance use and/or mental health services, which is consistent with prior research [27]. A third explanation may have to do with treatment effectiveness. Treatment effects can be difficult to determine in observational data [26] without detailed information about treatment modality, treatment response and the sequence of treatment, treatment adherence, and symptom severity, information that is often absent from epidemiologic surveys. There were likely a variety of different patterns of treatment persistence and discontinuity between waves 1 and 2, which could also impact symptom progression or remission during that time. Our study only included a dichotomous measure of any SU or MH treatment and, therefore, did not capture treatment type, quality, dose, frequency, adherence, or duration. This type of health service information would have important implications for the efficacy of the treatments the participants had received.

It is also possible that there were unmeasured confounders that contributed to these findings. For instance, while histories of mood, anxiety, and personality disorders were included in the propensity score model, we could not account for history of other mental illnesses, psychiatric symptom severity, or response to mental health treatment as that information was unavailable in the NESARC dataset. These unmeasured variables may have important implications for results, however, as research suggests that factors such as severe mental illness and psychiatric symptom severity impact treatment engagement and AUD prognosis [46].

While the focus of these analyses was on past or current female drinkers’ transitions across classes of alcohol involvement, it is notable that we found women who identified as Black, Hispanic, or other race or ethnicity were less likely to have received SU or MH treatment than White women, with particularly disparities noted between Black and White women. Racial disparities in access to behavioral healthcare have been well-documented [47–49]. While identifying as Asian, Black, or Hispanic is associated with lower lifetime prevalence of AUD [9], people with minoritized racial identities experience longer delays in initiating treatment [47] and experience higher rates of recurrence and persistence and greater impairment and consequences resulting from alcohol use [50]. Prior analyses have found that Black women have a longer time between initiating drinking and entering treatment than White women [28]. Additionally, women ages 50 years and older appeared to be underrepresented among those who received treatment. This is also consistent with prior research demonstrate that younger adults are more likely to use mental health services than middle-aged or older adults [51]. Results from the current study and prior work highlight the need for further research to understand and reduce disparities in access to care and in alcohol use related outcomes for racially minoritized women and for older women.

Our results should be interpreted in the context of limitations. First, SU and MH treatment data are based on participants’ retrospective self-report, which could potentially contribute to underreporting of treatment use. Second, as noted above, detailed information about the quality and quantity of treatment received was unavailable. Finally, the follow-up period in NESARC is three years; it is possible that a longer follow up period might yield different results. However, three years is longer than most clinical follow up studies and, as noted by the original NESARC researchers, a longer follow up period would have increased costs and made study retention more challenging [52]. Despite the limitations of the data, we chose to use NESARC data because NESARC waves 1 and 2 comprise the largest and most recent longitudinal mental health and substance use survey of the US general adult population, with detailed information about alcohol and other substance use as well as information on MH symptoms and SU and MH treatment. This dataset provided a large enough sample size to power LCA and LTA, to identify latent classes of alcohol involvement, transitions between classes, and the association between those transitions and receiving SU or MH treatment in usual care settings.

## Conclusion

Consistent with our hypotheses, women who received treatment were more likely to experience remitting alcohol use involvement across time. Future research should examine associations between treatment utilization and transitions across stages of alcohol involvement at additional time points and in more detail to better pinpoint when treatment began and ended and when alcohol use problems changed. While not the primary focus of our study, our findings indicated that there were significant racial and ethnic and age-related disparities in obtaining SU and MH treatment. With alcohol use and misuse increasing among women as well as among those from historically minoritized backgrounds [8], it may be particularly important for future research to examine predictors of treatment seeking and treatment efficacy within the context of intersectionality.

## Supporting information

S1 Table Latent class analysis indicators–alcohol abuse (4 criteria) and alcohol dependence (7 criteria) criteria from the alcohol use disorder and associated disabilities interview Schedule-IV. (DOCX)
