# Supplementary material for: Associations between mental health & substance use treatment and alcohol use progression and recovery among US women drinkers
Source: PLoS One. 2024 Jul 8;19(7):e0306820. doi: 10.1371/journal.pone.0306820 (PMC11230554; doi:10.1371/journal.pone.0306820)
Supplement: S1 Table — (DOCX) [file pone.0306820.s001.docx]

**S1 Table. Latent Class Analysis Indicators – Alcohol Abuse (4 criteria) and Alcohol Dependence (7 criteria) Criteria from the Alcohol Use Disorder and Associated Disabilities Interview Schedule-IV**(1, 2)

| **Disorder Category** | **Indicator (label given on the x axis of Figure 1)** |
| --- | --- |
| **Alcohol Abuse** | Drinking interferes with home/family, job or school (Major role failure) |
|  | Drinking increased chances of getting hurt (Hazardous drinking) |
|  | Had legal problems as a result of drinking (Legal problems) |
|  | Continued to drink even though it caused social problems (Social Problems) |
| **Alcohol Dependence** | Had to drink more to get desired effect/usual number of drinks have a lesser effect than they used to (Tolerance) |
|  | Experienced symptoms of withdrawal as alcohol effects were wearing off (Cut down) |
|  | Drank more or longer than intended (Larger amount) |
|  | Tried to drink less or stop drinking but couldn’t (Withdrawal) |
|  | Spent a lot of time drinking (Time spent) |
|  | Given up or reduced time spent in activities that are important or interesting (Give up) |
|  | Continued to drink even though it caused anxiety/depression or other health problems (Phys/Psych problems) |

References

1. American Psychiatric Association. Diagnostic and statistical manual of mental disorders: DSM-IV-TR. 4th ed., text revision ed. Washington, DC: American Psychiatric Association; 2000 2000. 943 p.

2. Grant BF, Dawson DA, Stinson FS, Chou PS, Kay W, Pickering R. The Alcohol Use Disorder and Associated Disabilities Interview Schedule-IV (AUDADIS-IV): Reliability of alcohol consumption, tobacco use, family history of depression and psychiatric diagnostic modules in a general population sample. Drug and Alcohol Dependence. 2003;71(1):7-16.
